# Supplementary figures and images for: Antigen presentation by clonally diverse CXCR5+ B cells to CD4 and CD8 T cells is associated with durable response to immune checkpoint inhibitors
Source: Front Immunol. 2023 Jun 26;14:1176994. doi: 10.3389/fimmu.2023.1176994 (PMC10330698; doi:10.3389/fimmu.2023.1176994)

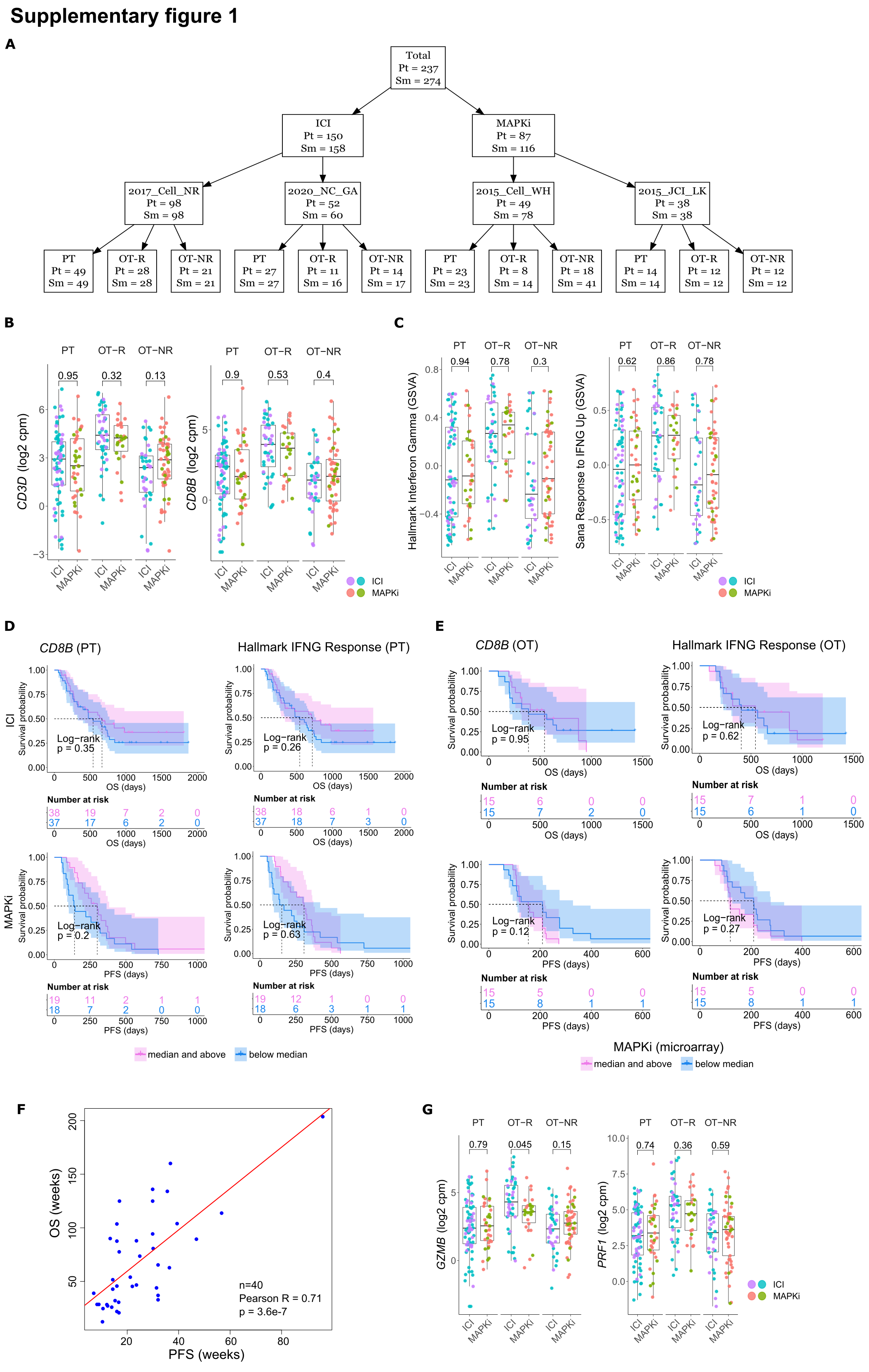

Supplement: Supplementary Figure 1 — Shared and therapy-specific transcriptomic changes after ICI or MAPKi therapy. (A) Schematic of the bulk RNA-seq data sets of ICI- and MAPKi-treated melanoma used in this study. (B, C) Normalized expression of T cell marker genes (CD3D, CD8B) (B) and GSVA gene set enrichment scores of interferon gamma gene sets from the Molecular Signature database (C) in the PT, OT-R and OT-NR samples of patients treated with ICI or MAPKi therapy (inter therapy comparison). (D) Kaplan-Meier survival curves of ICI- or MAPKi-treated melanoma patients stratified by either CD8B expression (left) or hallmark interferon gamma response gene set scores (right) in their PT tumors. (E) Kaplan-Meier survival curves of overall (top) or progression free survival (bottom) of MAPKi-treated melanoma patients stratified by either CD8B expression (left) or hallmark interferon gamma response gene set scores (right) in their OT tumors (two independent microarray datasets of MAPKi-treated melanoma patients). (F) Correlation between PFS and OS after MAPKi therapy across two separate melanoma microarray datasets. (G) Normalized expression of T cell cytotoxicity genes GZMB and PRF1 in the PT, OT-R and OT-NR samples of patients treated with ICI or MAPKi therapy. [file Image_1.tiff]

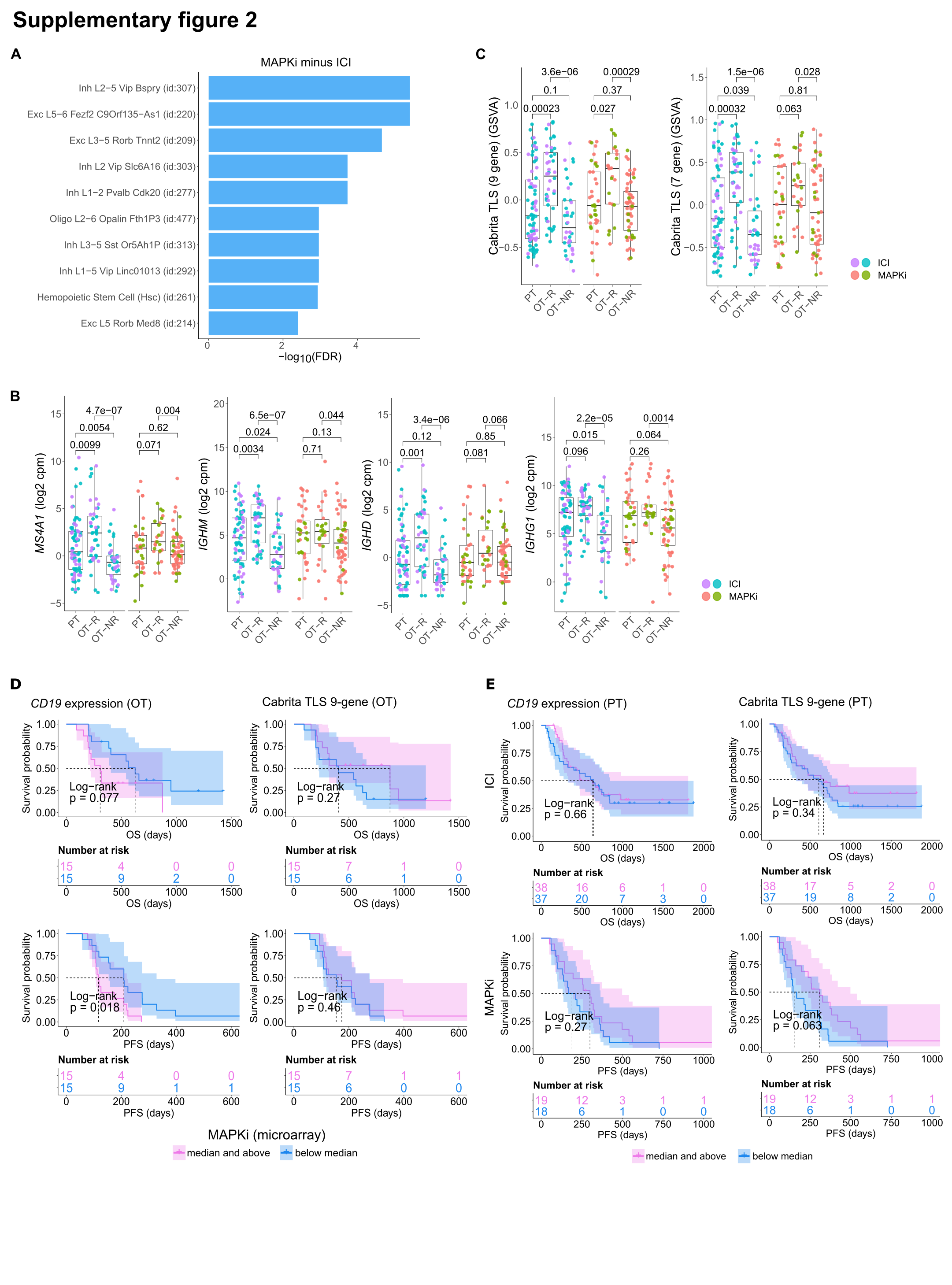

Supplement: Supplementary Figure 2 — Characterization of B cell and TLS-related gene expression changes after ICI and MAPKi therapy. (A) Enriched tissue specific gene sets in DEGs upregulated in MAPKi OT-R tumors with respect to ICI OT-R tumors (after adjustment by respective therapy group’s OT-NR tumors). (B) Normalized expression of the listed B cell-related genes among the PT, OT-R and OT-NR tumors in the ICI or MAPKi therapy group. (C) Enrichment scores of a recently published TLS 7-gene and 9-gene gene sets among the PT, OT-R and OT-NR tumors in the ICI or MAPKi therapy group. (D) Kaplan-Meier survival curves of overall (top) or progression free survival (bottom) of MAPKi-treated melanoma patients stratified by either CD19 expression (left) or TLS gene set enrichment score (right) in their OT tumors. (E) Kaplan-Meier survival curves of ICI- or MAPKi-treated melanoma patients stratified by either CD19 expression (left) or TLS gene set enrichment score (right) in their PT tumors. [file Image_2.tiff]

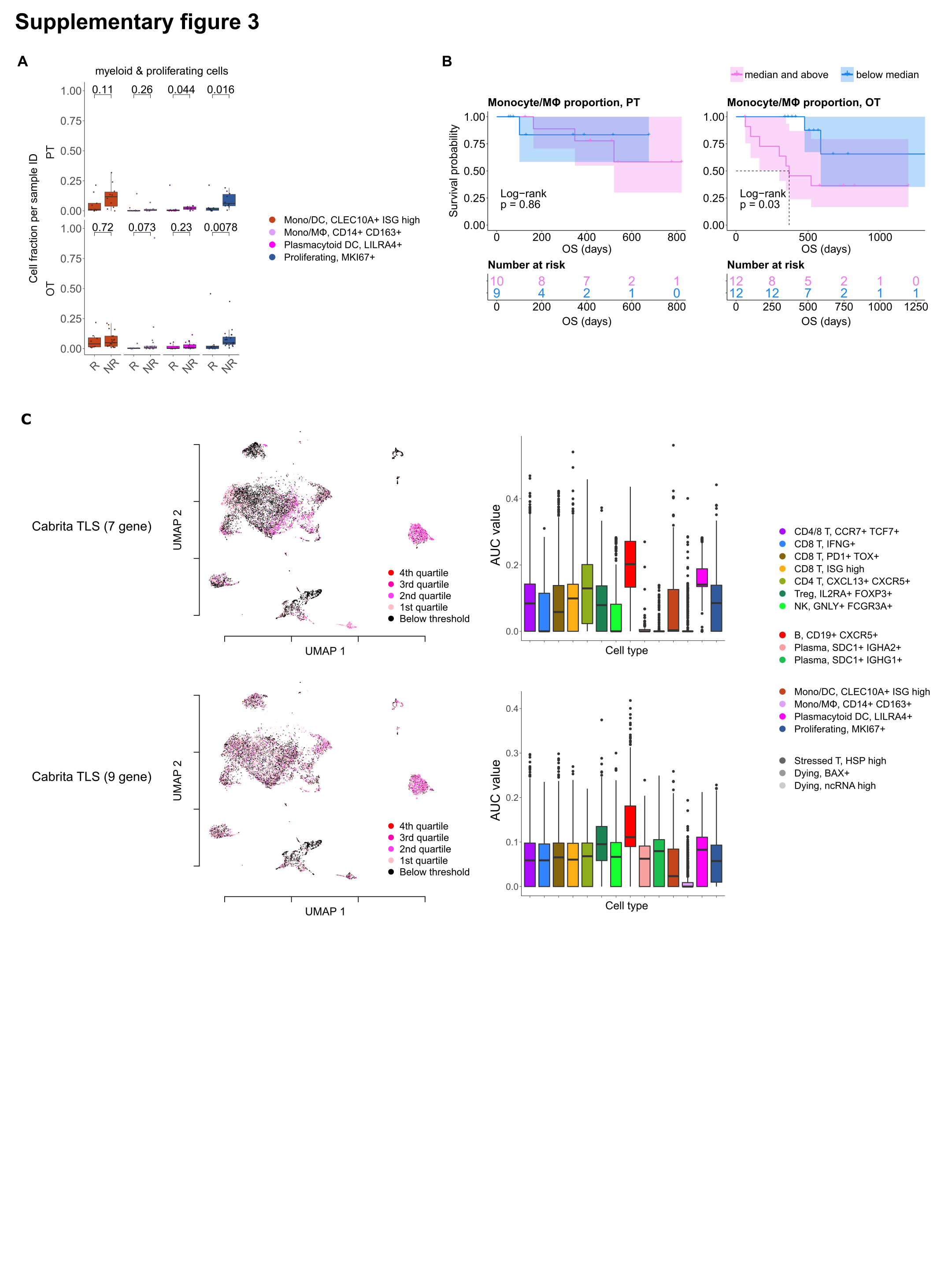

Supplement: Supplementary Figure 3 — Single cell analysis of ICI-treated melanoma. (A) The fraction of the myeloid and proliferating cell populations in stratified by response vs. non-response to ICI in PT (top) and OT tumors (bottom). (B) Kaplan-Meier survival curves of ICI-treated patients stratified by the proportion of monocyte/macrophages within either their PT (left) or OT tumors (right). (C) Single cell-based gene set enrichment score of the TLS 7-gene and 9-gene signatures projected on the UMAP (left) or presented in boxplot across all cell types (right). The scores were separated into quartiles to assist the visualization of high and low gene set enrichments in different cell populations. [file Image_3.tiff]

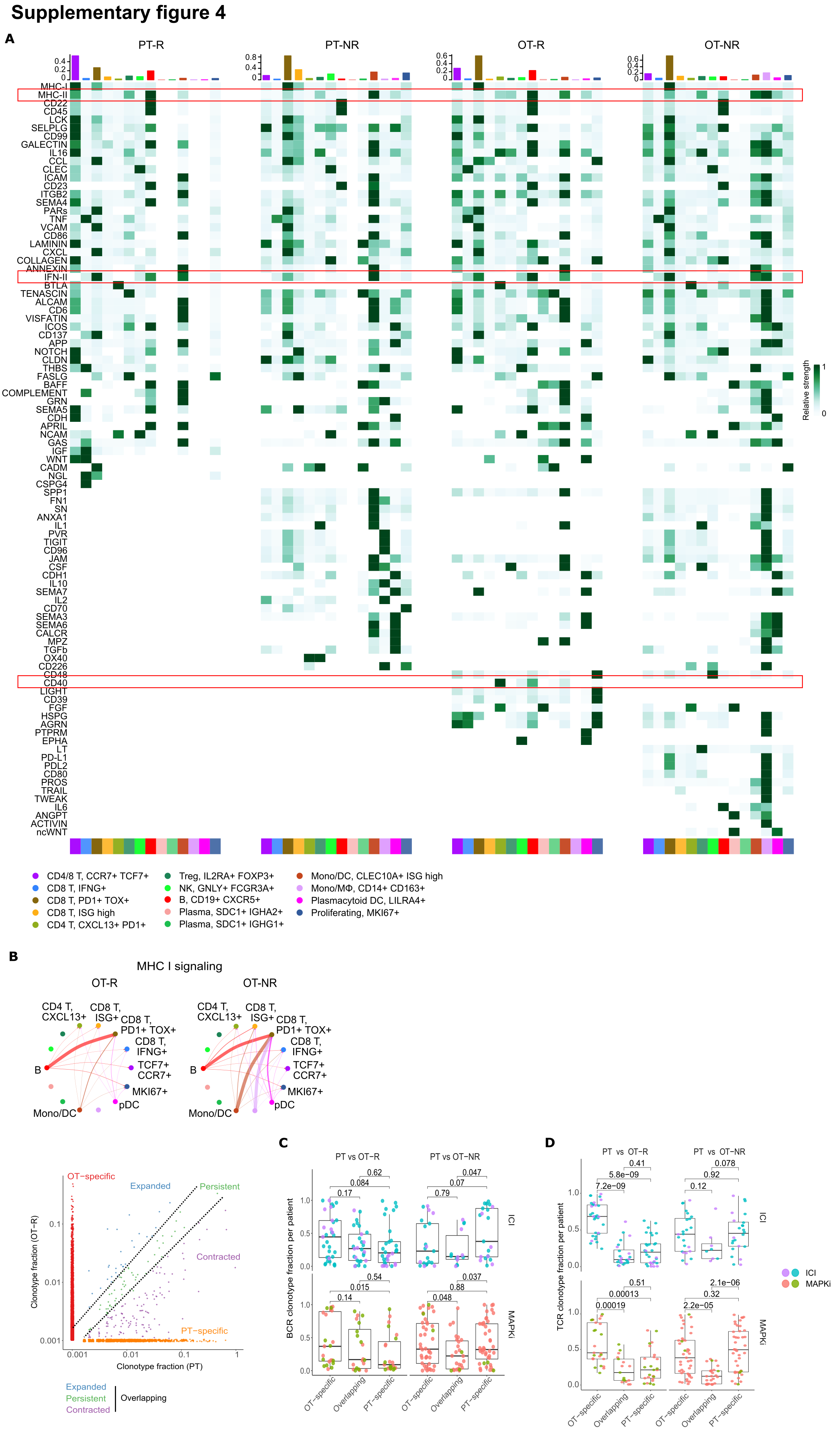

Supplement: Supplementary Figure 4 — B cell-associated antigen presentation and clonotype analyses. (A) Inferred cell-cell communication among intratumoral immune populations of pre- and post-ICI treated melanoma across curated signaling pathways in CellChat. The interactions are grouped based on response vs. no-response to ICI in either PT or OT tumors. (B) Predicted enrichment of cell-to-cell interaction through MHC I antigen presentation pathway. (C, D) Change in BCR (C) or TCR (D) clonal fraction in grouped by clones found only in the OT sample (OT-specific), only in the PT (PT-specific) and both in the PT and OT samples (overlapping, see illustration on the left). These fractions are calculated with respect to the union of all BCR/TCR clones found in the PT and OT samples of each patient; this analysis is done only on patients with PT and OT tumors. [file Image_4.tiff]

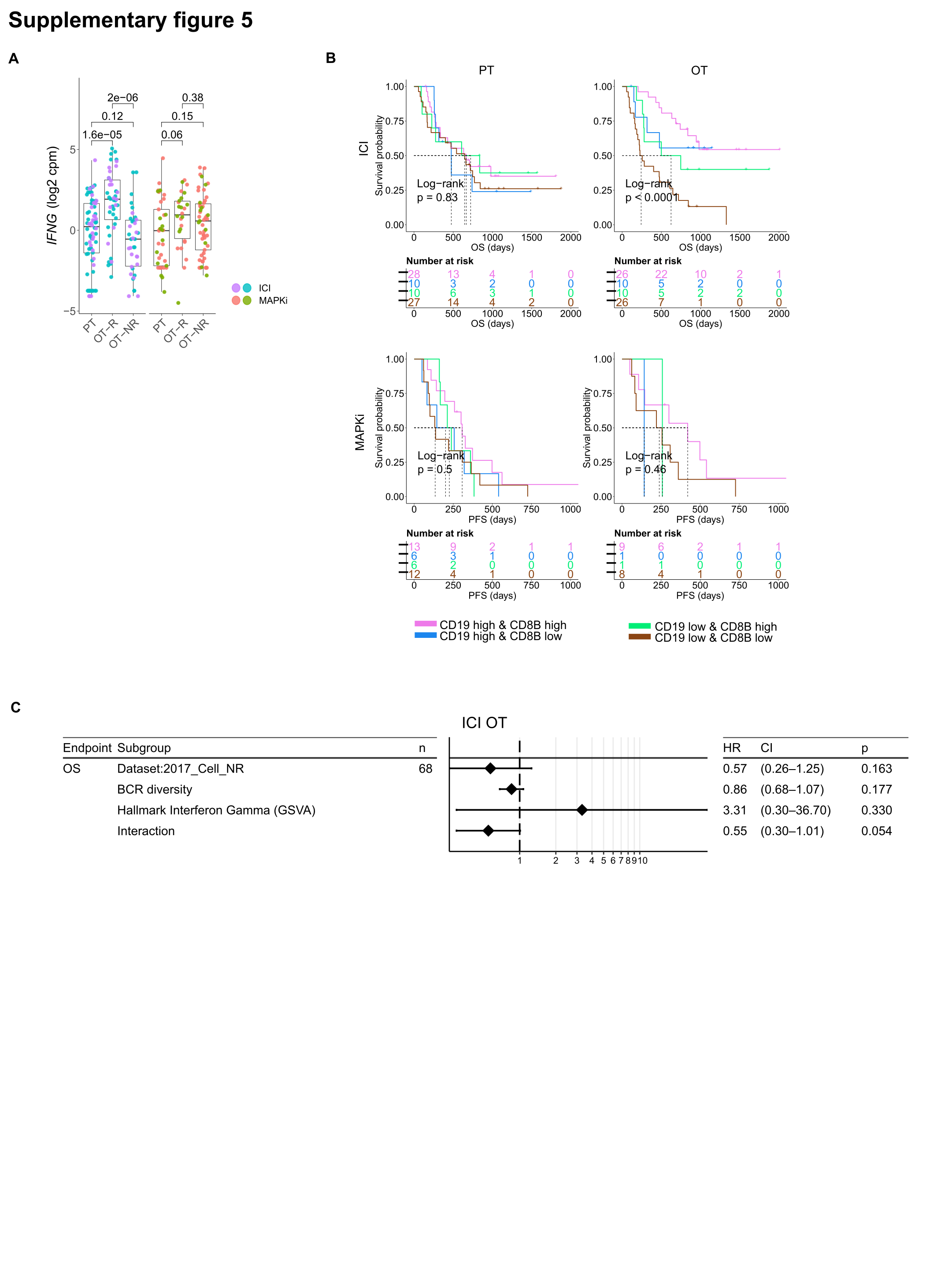

Supplement: Supplementary Figure 5 — Multivariate analysis of survival examining the association among T cell and B cell related variables to patient OS after ICI therapy. (A) Normalized bulk RNA-seq expression of IFNG in the PT, OT-R and OT-NR samples of patients treated with ICI or MAPKi therapy. (B) Kaplan-Meier survival curves of patients stratified by normalized expressions of CD8B and CD19 in either PT or OT tumors of the ICI or MAPKi therapy group. (C) Multivariate Cox proportional hazards analysis assessing the hazard ratios of BCR diversity, hallmark interferon gamma gene set score, and their interaction in ICI OT tumor samples. [file Image_5.tiff]
